# The “Central Vein Sign” on T2*-weighted Images as a Diagnostic Tool in Multiple Sclerosis: A Systematic Review and Meta-analysis using Individual Patient Data

**DOI:** 10.1038/s41598-019-54583-3

**Published:** 2019-12-03

**Authors:** Chong Hyun Suh, Sang Joon Kim, Seung Chai Jung, Choong Gon Choi, Ho Sung Kim

**Affiliations:** Department of Radiology and Research Institute of Radiology, University of Ulsan College of Medicine, Asan Medical Center, 86 Asanbyeongwon-Gil, Songpa-Gu, Seoul, 05505 Republic of Korea

**Keywords:** Diagnostic markers, Multiple sclerosis

## Abstract

We aimed to evaluate the pooled incidence of central vein sign on T2*-weighted images from patients with multiple sclerosis (MS), and to determine the diagnostic performance of this central vein sign for differentiating MS from other white matter lesions and provide an optimal cut-off value. A computerized systematic search of the literature in PUBMED and EMBASE was conducted up to December 14, 2018. Original articles investigating central vein sign on T2*-weighted images of patients with MS were selected. The pooled incidence was obtained using random-effects model. The pooled sensitivity and specificity were obtained using a bivariate random-effects model. An optimal cut-off value for the proportion of lesions with a central vein sign was calculated from those studies providing individual patient data. Twenty-one eligible articles covering 501 patients with MS were included. The pooled incidence of central vein sign at the level of individual lesion in patients with MS was 74% (95% CI, 65–82%). The pooled sensitivity and pooled specificity for the diagnostic performance of the central vein sign were 98% (95% CI, 92–100%) and 97% (95% CI, 91–99%), respectively. The area under the HSROC curve was 1.00 (95% CI, 0.99–1.00). The optimal cut-off value for the proportion of lesions with a central vein sign was found to be 45%. Although various T2*-weighted images have been used across studies, the current evidence supports the use of the central vein sign on T2*-weighted images to differentiate MS from other white matter lesions.

## Introduction

The accurate diagnosis of multiple sclerosis (MS) is clinically important to avoid inappropriate management or unnecessary invasive biopsy. MRI is the most commonly performed investigation able to support a clinical diagnosis of MS, and MRI might be useful for ruling out MS-mimicking pathologies^1,2^. However, even though the 2017 McDonald criteria have been published, there are still challenging cases and misdiagnoses, which are prevalent problems in MS^3,4^. Therefore, there is still a need for more accurate MRI criteria that can exclude other MS-mimicking white matter lesions.

The “central vein sign” which is considered to be a MRI-detectable central vein inside white matter lesion identified as a hypointensity relative to the surrounding lesion on T2*-weighted images, has been introduced as a biomarker of inflammatory demyelination^1,5^. This central vein sign has been investigated in various neurological conditions, including MS, cerebral small vessel disease, neuromyelitis optica spectrum disorder (NMOSD), inflammatory vasculopathies, and migraine, and evidence has accumulated that the central vein sign may allow the accurate differentiation of MS from other white matter lesions^6–26^.

The North American Imaging in Multiple Sclerosis Cooperative mentioned that, as the differential diagnosis of MS is broad, the pooling of data from multiple centers would be a realistic strategy for conducting a systematic and well-powered evaluation of the central vein sign on T2*-weighted images^5^. To our knowledge, the incidence of central vein sign on T2*-weighted images from patients with MS, and its diagnostic performance for differentiating MS from other white matter lesions, have not yet been systematically reviewed. Therefore, we performed a systematic review and meta-analysis to evaluate the pooled incidence of central vein sign on T2*-weighted imaging of patients with MS. In addition, we aimed to determine the diagnostic performance of the central vein sign for differentiating MS from other white matter lesions and provide an optimal cut-off value for this differentiation.

## Materials and Methods

This systematic review and meta-analysis was conducted according to the Preferred Reporting Items for a Systematic Review and Meta-analysis of Diagnostic Test Accuracy Studies (PRISMA-DTA) statement^27,28^.

### Literature Search

A computerized systematic search of the literature in PUBMED and EMBASE was conducted to find published original articles investigating the central vein sign on T2*-weighted imaging of patients with MS. The search term combined synonyms of “multiple sclerosis” and “central vein” as follows: ((“multiple sclerosis”)) AND ((“central vein”) OR (vein in lesion) OR (perivenular)). The databases were searched for articles published up to December 14, 2018. The search was restricted to English-language publications. A manual search was also performed to find additional relevant articles. EndNote X8 was used for literature handling.

### Eligibility criteria

Studies were selected if all of the following inclusion criteria were met: (1) patients with MS; (2) patients underwent MRI including T2*-weighted images; and (3) provision of sufficient information for the incidence of central vein sign on T2*-weighted images or the reconstruction of 2 × 2 tables for determination of the diagnostic performance of central vein sign for diagnosis of MS.

Studies were excluded if any of the following exclusion criteria were satisfied: (1) conference abstracts; (2) review articles; (3) case reports or case series including fewer than five patients; (4) letters, editorials, and short surveys; (5) studies with a partially overlapping patient cohort, and (6) animal studies. For studies with a partially overlapping study population, the study including the largest number of patients was selected. Authors of potentially eligible articles that did not provide sufficient information were contacted for the provision of further data.

### Data extraction and quality assessment

The incidence of central vein sign on T2*-weighted images from patients with MS and the diagnostic performance of the central vein sign for differentiating MS from other white matter lesions were extracted from the eligible articles. Central vein sign on T2*-weighted imaging was defined as follows: (1) the vein should appear as a thin line or dot; (2) when technically possible, the vein should be visualized in at least two perpendicular planes; and (3) the vein can run partially or entirely through the lesion, but must be located centrally, regardless of the lesion’s shape^5^. Two by two tables (true positive, false positive, false negative, true negative) for determination of the diagnostic performance of the central vein sign for differentiating MS from other white matter lesions such as small vessel disease, CNS inflammatory vasculopathies, or NMOSD were also constructed. If the diagnostic performances of multiple MRI sequences were separately evaluated, the results with the highest performance were selected. If a two by two table could not be acquired, the authors were contacted for provision of further data by E-mail.

The following information was extracted from the eligible studies: (1) the institution, the study period, study design (retrospective or prospective design), consecutive or non-consecutive patient enrollment, and the reference standard; (2) the number of MS patients, mean age, age range, and female to male ratio; (3) the magnetic field strength of the scanner, scanner manufacturer, scanner model, MRI sequence, and cut-off values for the proportion of lesions with central vein sign used to diagnose MS; and (4) the number of MRI readers, and blindness to the reference standard.

Quality assessment was performed using the Quality Assessment of Diagnostic Accuracy Studies-2 (QUADAS-2) criteria^29^. The literature search, study selection, data extraction, and quality assessment were performed by two reviewers (C.H.S., S.J.K.).

### Statistical analyses

The pooled incidence of central vein sign on T2*-weighted images of MS was calculated with the inverse variance method for calculating weights and the DerSimonian-Liard random-effects model^30–32^. Heterogeneity was assessed by Higgins inconsistency index (I^2^) test, with values greater than 50% taken as indicating substantial heterogeneity^33^. Publication bias was assessed by a funnel plot, and the statistical significance was assessed by Egger’s test^34^. Meta-regression was conducted to explain the effects of study heterogeneity. The following covariates were considered: (1) study design (prospective study vs. other); (2) MRI sequence (studies including FLAIR* [combined FLAIR and T2*-weighted images]^35^ vs. other); (3) reader (radiologist vs. other); (4) reader blindness to the reference standard; and (5) patient age (age ≤41 [median value] vs. age >41). Subgroup analyses according to the strength of the MRI scanner (7, 3, and 1.5-Tesla) were also performed.

The pooled sensitivity and specificity and their 95% confidence intervals [CI] for the diagnostic performance of central vein sign on T2*-weighted images for differentiating MS from other white matter lesions were calculated using a bivariate random-effects model^30–32^. A coupled forest plot of sensitivity and specificity and a hierarchical summary receiver operating characteristic (HSROC) curve with 95% confidence and prediction regions were plotted. Heterogeneity was assessed by the following methods: (1) Cochran’s Q-test (*p* < 0.05 indicating the presence of heterogeneity); (2) Higgins I^2^ test (a value >50% indicating the presence of heterogeneity);^33^ (3) visual assessment of the coupled forest plot for the presence of a threshold effect, i.e., a positive correlation between sensitivity and false positive rate; and (4) the Spearman correlation coefficient between sensitivity and false positive rate (a value >0.6 indicating a threshold effect)^36^. Publication bias was assessed by Deeks’ funnel plot, with the statistical significance being assessed by Deeks’ asymmetry test^37^. A meta-regression was conducted to explain the effects of study heterogeneity, with the following covariates being utilized for the bivariate meta-regression model: (1) study design (prospective study vs. other); (2) MRI sequence (studies including FLAIR*^35^ vs. other); (3) reader (radiologist vs. other); (4) reader blindness to the reference standard; and (5) patient age (age ≤41 [median value] vs. age >41). Subgroup analysis was conducted on those studies using a proportion of lesions with central vein sign as a cut-off value.

An optimal cut-off value for the proportion of lesions with central vein sign was calculated from those studies providing individual patient data. The individual patient data were extracted from the articles, and when not reported, Plot Digitizer 2.6.8 (plotdigitizer.sourceforge.net) was used to estimate the data from plots indicating the proportion of lesions with central vein sign. The sensitivity and specificity of the central vein sign and the corresponding cut-off value for the proportion of lesions with a central vein sign were estimated using the Youden index. The Youden index is defined as sensitivity + specificity – 1, with it having a minimum value of −1 and a maximum value of + 1, with a value of + 1 indicating the optimal value for an algorithm^38^.

Statistical analyses were performed by one reviewer (C.H.S., with 6 years of experience in performing systematic reviews and meta-analysis) using the “metafor” and “mada” packages in R v.3.4.1 (R Foundation for Statistical Computing, Austria), and the “metandi” and “midas” modules in STATA 15.0 (StataCorp, College Station, USA).

## Results

### Literature search

**Figure 1:**
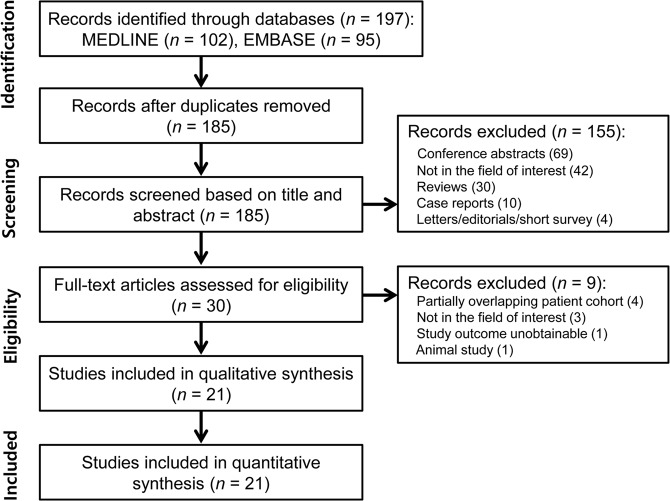
Flow diagram for the literature selection process.

The details of the study selection process are illustrated in Fig. 1 and Supplementary materials. Finally, 21 eligible articles encompassing 501 patients with MS were included in the analyses^6–26^.

### Characteristics of the included studies

The characteristics of the eligible studies are shown in Table 1. Six studies had a prospective design^8,10,15,18,21,23^ and six studies had a retrospective design^7,14,16,17,22,25^, with the other studies not reporting the design. Patient enrollment was conducted in a consecutive fashion in only three studies^18,20,25^, with this detail not being reported in the others. The eligible studies included numbers of MS patients ranging from 8 to 68. Fifteen studies^6–10,12,14–17,20,21,23–25^ used the revised 2010 McDonald criteria^39^, two studies^13,26^ used the revised 2005 McDonald criteria^40^, and the other four studies^11,18,19,22^ did not mention the criteria used to diagnose MS.

**Table 1:** Characteristics of the eligible studies.

Author (year of publication)	Duration of patient recruitment	Institution	Study design	MS patients (*n*)	Mean age (years)	Female:Male
Al-Zandi SH, *et al*.^6^ (2018)	2016.4–2017.3	Al-Imamain Al-Kadhymain Medical City, Iraq	NA	30	40.8 (22–58)	7:23
Campion T, *et al*.^7^ (2017)	NA	The Royal London Hospital, UK	retrospective	25	41	14:11
Cortese R, *et al*.^8^ (2018)	NA	Walton Centre and the National Hospital for Neurology and Neurosurgery, UK	prospective	18	41.8	4:14
Darwish EAF, *et al*.^9^ (2018)	NA	Ain Shams University, Egypt	NA	9	33 (26–45)	2:7
Gabr RE, *et al*.^10^ (2018)	NA	University of Texas Health Science Center at Houston, USA	prospective	15	43 (26–62)	6:9
Gaitan MI, *et al*.^11^ (2013)	NA	National Institutes of Health, USA	NA	8	37.1	2:6
George IC, *et al*.^12^ (2016)	NA	National Institutes of Health, USA	NA	68	46	36:32
Grabner G, *et al*.^13^ (2011)	NA	Medical University of Vienna, Austria	NA	8	NA	NA
Hosseini Z, *et al*.^14^ (2018)	NA	University of Western Ontario, Canada	retrospective	17	39.4 (26–46)	6:11
Kau T, *et al*.^15^ (2013)	NA	Klinikum Klagenfurt, Austria	prospective	5	47 (20–57)	1:4
Lamot U, *et al*.^16^ (2017)	NA	University Medical Centre Ljubljana, Slovenia	retrospective	34	39.6 (21–66)	9:25
Lane JI, *et al*.^17^ (2015)	18 months	Mayo Clinic, USA	retrospective	21	46	5:16
Lummel N, *et al*.^18^ (2011)	NA	University of Munich, Germany	prospective	15	48.4	3:12
Luo J, *et al*.^19^ (2014)	NA	Washington University, USA	NA	30	51.5 (27–70)	14:16
Maggi P, *et al*.^20^ (2018)	2015.1–2017.6	Multicenter (four academic research hospitals)	NA	52	41 (20–65)	18:34
Mistry N, *et al*.^21^ (2016)	NA	Nottingham University Hospitals NHS Trust, UK	prospective	23	45.5 (25–66)	13:10
Oztoprak B, *et al*.^22^ (2016)	2013.9–2014.9	Cumhuriyet University School of Medicine, Turkey	retrospective	38	34.3	5:33
Sinnecker T, *et al*.^23^ (2012)	NA	NeuroCure Clinical Research Center, Charité – Universitaetsmedizin Berlin, Germany	prospective	18	41 (27–53)	7:11
Solomon AJ, *et al*.^24^ (2018)	NA	University of Vermont, USA	NA	20	43.5	2:18
Sparacia G, *et al*.^25^ (2018)	2016.12–2017.4	University of Palermo, Italy	retrospective	19	36.9 (19–53)	9:10
Tallantyre EC, *et al*.^26^ (2011)	2007.8–2009.8	Nottingham University Hospitals NHS Trust, UK	NA	28	46.5	16:12

MS = multiple sclerosis, NA = not available.

The MRI characteristics are shown in Table 2. Twelve studies used a 3-Tesla scanner^6–10,12,15,16,18,19,21,24^, four studies used a 7-Tesla scanner^11,14,23,26^, three studies used a 1.5-Tesla scanner^17,22,25^, one study used either 7- or 3-Tesla scanners^13^, and one study used either 3- or 1.5-Tesla scanners^20^. All studies used T2*-based MRI sequences; eight studies used FLAIR and susceptibility-weighted imaging (SWI)^9,13–18,22^, five studies used FLAIR*^7,10,12,20,24^, four studies used T2*-weighted images^11,21,23,26^, three studies used SWI^6,19,25^, and one study used proton density-weighted imaging, T2-weighted imaging, and SWI^8^.

**Table 2:** MRI characteristics of the eligible studies.

	Magnetic field strength	Scanner model, manufacturer	MRI sequences	MRI readers	MRI reader blindness to the reference standard
Al-Zandi SH, *et al*.^6^ (2018)	3 T	Achieva, Philips	SWI	2 radiologists	NA
Campion T, *et al*.^7^ (2017)	3 T	Achieva TX, Philips	FLAIR*	2 neuroradiologists, 1 radiology resident	yes
Cortese R, *et al*.^8^ (2018)	3 T	Achieva, Philips	PD, T2WI, SWI	2 neuroradiologists	yes
Darwish EAF, *et al*.^9^ (2018)	3 T	Magnetom Skyra, Siemens	FLAIR, SWI	2 neuroradiologists	yes
Gabr RE, *et al*.^10^ (2018)	3 T	Ingenia, Philips	FLAIR*	1 neuroradiologist	NA
Gaitan MI, *et al*.^11^ (2013)	7 T	NA, Siemens	T2*-weighted images	NA	NA
George IC, *et al*.^12^ (2016)	3 T	NA, Philips	T2-FLAIR + FLAIR*	2 neurologists	yes
Grabner G, *et al*.^13^ (2011)	3 T, 7 T	Tim Trio, 7 T system, Siemens	FLAIR (3 T), SWI (7 T)	1 radiologist	NA
Hosseini Z, *et al*.^14^ (2018)	7 T	NA, Agilent Technologies	FLAIR, SWI	1 neuroradiologist, 1 radiology resident	NA
Kau T, *et al*.^15^ (2013)	3 T	Achieva, Philips	FLAIR, SWI	2 readers	yes
Lamot U, *et al*.^16^ (2017)	3 T	Magnetom Trio, Siemens	FLAIR, T2WI, SWI	2 neuroradiologists	yes
Lane JI, *et al*.^17^ (2015)	1.5 T	Avanto or Espree, Siemens	FLAIR, SWI	3 neuroradiologists	yes
Lummel N, *et al*.^18^ (2011)	3 T	Signa HDxt, GE	FLAIR, SWAN	2 neuroradiologists	yes
Luo J, *et al*.^19^ (2014)	3 T	Trio, Siemens	SWI	NA	NA
Maggi P, *et al*.^20^ (2018)	3 T or 1.5 T	Best or Achieva, Philips	FLAIR*	2 neurologists	yes
Mistry N, *et al*.^21^ (2016)	3 T	Achieva, Philips	T2*-weighted images	1 neurologist, 1 neuroradiologist	yes
Oztoprak B, *et al*.^22^ (2016)	1.5 T	Magnetom Aera, Siemens	FLAIR, SWI	2 radiologists	NA
Sinnecker T, *et al*.^23^ (2012)	7 T	Magnetom, Siemens	T2*-weighted images	1 neuroradiologist, 1 trained observer	yes
Solomon AJ, *et al*.^24^ (2018)	3 T	NA, Philips	FLAIR*	3 neurologists	yes
Sparacia G, *et al*.^25^ (2018)	1.5 T	Achieva, Philips	SWI	2 neuroradiologists	yes
Tallantyre EC, *et al*.^26^ (2011)	7 T	Achieva, Philips	T2*-weighted images	1 primary observer	yes

SWI = susceptibility-weighted imaging, NA = not available, SWAN = susceptibility-weighted angiography.

### Quality assessment

The quality of the 21 eligible studies was considered as moderate, with more than four of the seven domains being satisfied (Supplementary Fig. 1). The details of the quality assessment are described in Supplementary materials.

### Incidence of the central vein sign on T2*-weighted imaging of patients with MS

**Figure 2:**
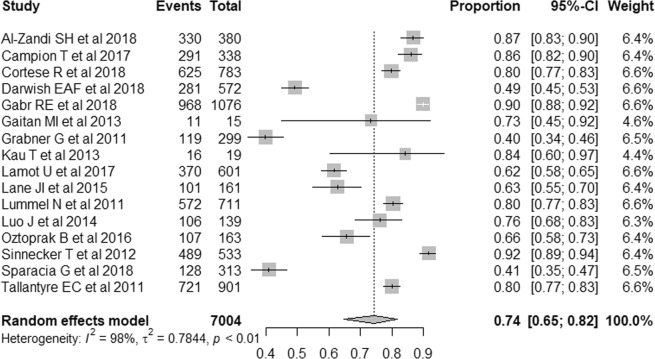
Forest plots showing the pooled incidence of central vein sign on T2*-weighted images in patients with MS. Numbers are estimates with 95% confidence intervals [CI] in parentheses.

Sixteen original articles evaluated the incidence of central vein sign on T2*-weighted images of patients with MS^6–11,13,15–19,22,23,25,26^. The individual incidences of central vein sign on T2*-weighted images at the level of individual lesion varied from 40% to 92%, while the pooled incidence of central vein sign on T2*-weighted images was 74% (95% CI, 65–82%; Fig. 2). Heterogeneity was present among these values (*I*^2^ = 98%). A meta-regression was performed to explore the effects of heterogeneity, and among the various covariates analyzed, the study design showed statistical significance (*p* = 0.01). Prospective studies showed a significantly higher pooled incidence of central vein sign on T2*-weighted images (86%; 95% CI, 80–91%) than retrospective studies (67%; 95% CI, 56–77%). Other covariates including MRI sequence (*p* = 0.08), reader (*p* = 0.51), reader blindness to the reference standard (*p* = 0.93), and age (*p* = 0.31) did not show statistically significant differences. There was no publication bias (*p* = 0.63; Supplementary Fig. 2).

In the subgroup analyses, the pooled incidence of central vein sign on T2*-weighted images was 84% (95% CI, 70–93%) in studies using 7-Tesla, 79% (95% CI, 68–87%) in studies using 3-Tesla, and 56% (95% CI, 39–72%) in studies using 1.5-Tesla. Although there were no statistical differences between 7-Tesla and 3-Tesla (*p* = 0.53), and between 3-Tesla and 1.5-Tesla (*p* = 0.05), there was a statistical difference between 7-Tesla and 1.5-Tesla (*p* = 0.01).

Eleven original articles evaluated the incidence of central vein sign on T2*-weighted images of patients with non-MS^6–9,15–18,23,25,26^. The pooled incidence of central vein sign on T2*-weighted images was 33% (95% CI, 18–52%). Heterogeneity was present (I^2^ = 99%). In the subgroup analyses, the pooled incidences of central vein sign were 26% (95% CI, 13–44%) in studies using 7-Tesla and 38% (95% CI, 18–63%) in studies using 3-Tesla. There were no statistical differences between 7-Tesla and 3-Tesla (*p* = 0.59). The pooled incidences of central vein sign were 36% (95% CI, 16–63%) in studies included patients with small vessel disease and 33% (95% CI, 28–38%) in studies included patients with NMOSD. There were no statistical differences between small vessel disease and NMOSD (*p* = 0. 91).

### Diagnostic performance of the central vein sign on T2*-weighted Images for diagnosis of MS

Twelve original articles evaluated the overall diagnostic performance of the central vein sign on T2*-weighted images for differentiating MS from other white matter lesions^6–9,12,14,15,20,21,23,24,26^. Four studies included patients with small vessel disease as a comparison group^6,7,21,26^, two studies included patients with NMOSD^8,23^, two studies included patients with CNS inflammatory vasculopathies^9,20^, two studies included healthy controls^12,14^, one study included non-MS white matter lesions^15^, and one study included patients with migraine who had been erroneously diagnosed with MS^24^.

**Figure 3:**
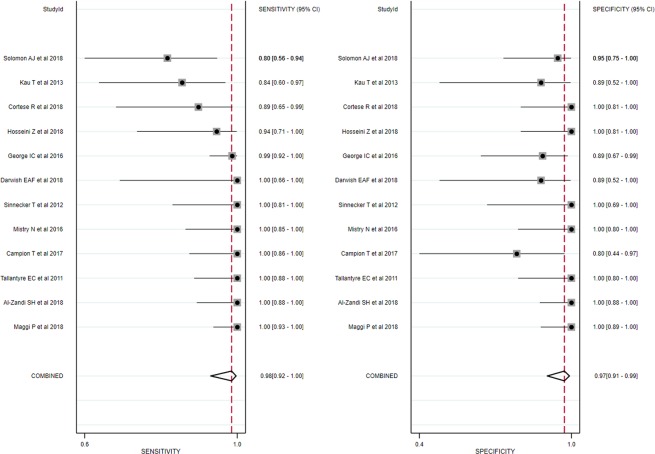
Coupled forest plots of the sensitivity and specificity of the central vein sign on T2*-weighted images for differentiating MS from other white matter lesions. Numbers are estimates with 95% confidence intervals [CI] in parentheses.

**Figure 4:**
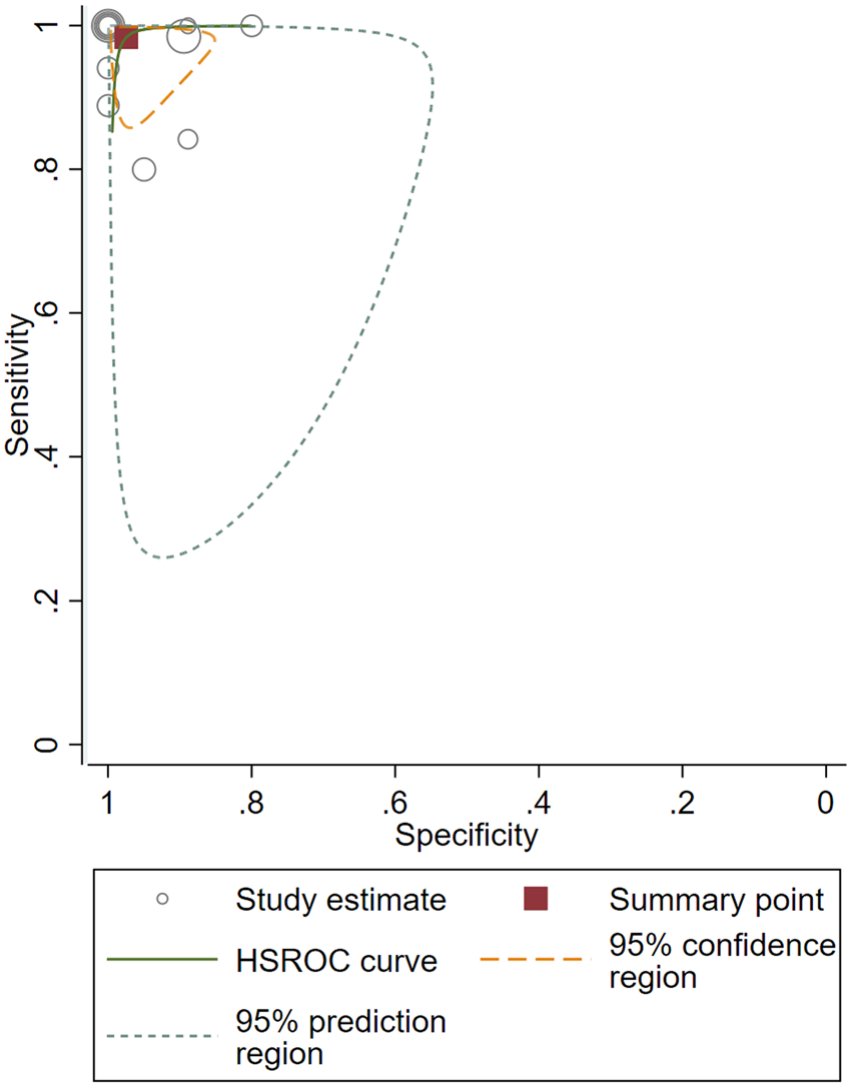
Hierarchical summary receiver operating characteristic (HSROC) curve of the diagnostic performance of the central vein sign on T2*-weighted images for differentiating MS from other white matter lesions.

Ten of the twelve studies used a cut-off parameter based on the proportion of lesions with central vein sign on T2*-weighted images^6–9,12,14,20,21,23,26^, and the patients with MS showed significantly higher proportions of lesions with central vein sign than did the patients with other white matter lesions. One study used just the presence of a central vein sign^15^, and one study used a simplified three-lesion algorithm^24^. The individual sensitivities and specificities both varied from 80% to 100%. The pooled sensitivity was 98% (95% CI, 92–100%), and the pooled specificity was 97% (95% CI, 91–99%; Fig. 3). The area under the HSROC curve was 1.00 (95% CI, 0.99–1.00; Fig. 4).

Both the Q-test (Q = 2.636, *p* = 0.13) and the Higgins I^2^ statistic (*I*^2^ = 24%) demonstrated that the possibility of heterogeneity was low across the studies. The coupled forest plot revealed no evidence of a threshold effect (Fig. 3), and the Spearman correlation coefficient was −0.092 (95% CI, −0.632–0.509), also indicating no threshold effect. The Deeks’ funnel plot demonstrated that publication bias was present (*p* < 0.01; Supplementary Fig. 3).

In the meta-regression, none of the covariates, including study design (*p* = 0.28), MRI sequence (*p* = 0.88), reader (*p* = 0.22), reader blindness to the reference standard (*p* = 0.46), and age (*p* = 0.36) significantly affected the heterogeneity. In the subgroup analysis, studies using a proportion of lesions with a central vein sign as a cut-off value^6–9,12,14,20,21,23,26^ also showed high sensitivity (99% [95% CI, 95–100%]) and specificity (99% [95% CI, 89–100%]), with the area under the HSROC curve being 0.99 (95% CI, 0.99–1.00). The Q-test (Q = 2.215, *p = *0.17) and the Higgins I^2^ statistic (*I*^2^ = 10%) demonstrated that the possibility of heterogeneity was low.

### Diagnostic performance using individual patient data

Eight studies provided individual patient data, including the proportion of lesions with a central vein sign on T2*-weighted images used for differentiating MS from other white matter lesions^7–9,14,20,21,23,26^. These studies included a total of 245 patients, with 177 patients (72.2%) having MS. Three studies had a prospective design^8,21,23^ and five studies had a retrospective design^7,9,14,20,26^. Four studies used a 3-Tesla scanner^7–9,21^, three studies used a 7-Tesla scanner^14,23,26^, and one study used either 3- or 1.5-Tesla^20^. Three studies used T2*-weighted images^21,23,26^, two studies used FLAIR and SWI^9,14^, two studies used FLAIR*^7,20^, and one study used proton density-weighted imaging, T2-weighted imaging, and SWI^8^. Three studies included patients with small vessel disease as a comparison group^7,21,26^, two studies included patients with NMOSD^8,23^, two studies included patients with CNS inflammatory vasculopathies^9,20^, and two studies included healthy controls^14^.

**Figure 5:**
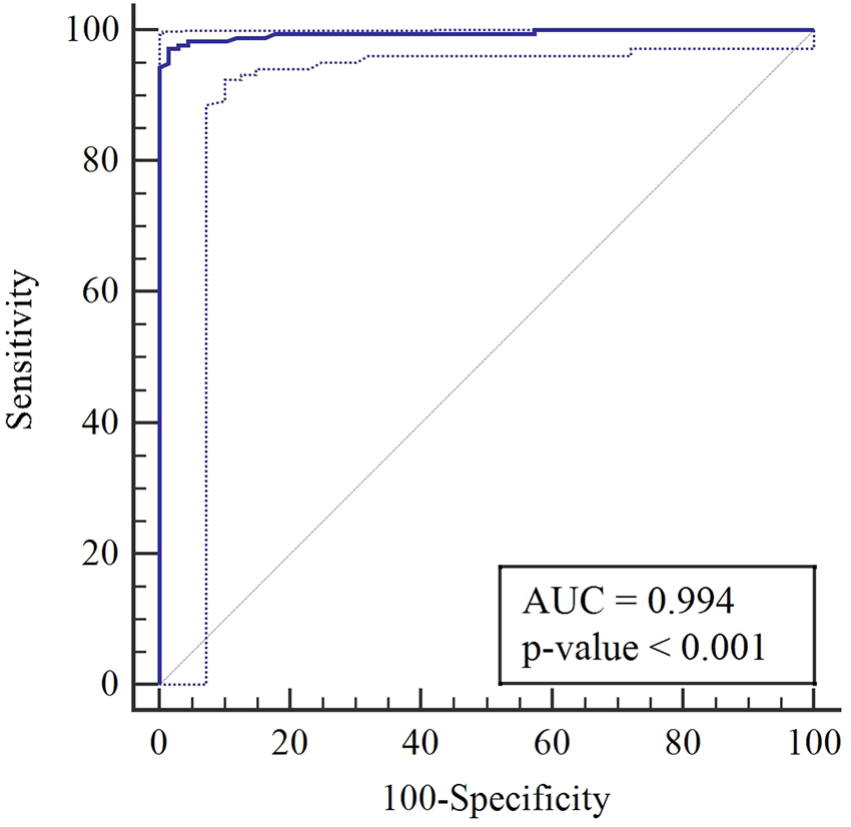
Receiver operating characteristic curve of the diagnostic performance of central vein sign on T2*-weighted images for differentiating MS from other white matter lesions according to individual patient data.

The individual cut-off values ranged from 30% to 67%, with a median value of 45%. The area under the ROC curve of the proportion of lesions with central vein sign for the diagnosis of MS was 0.994 (95% CI, 0.975–1.000; Fig. 5). The optimal cut-off value was 45% using the Youden index, resulting in a sensitivity of 97% (95% CI, 94–99%) and specificity of 99% (95% CI, 92–100%).

## Discussion

The current study revealed a high incidence (74%) of central vein sign on T2*-weighted images of patients with MS, and also revealed that the central vein sign has excellent diagnostic performance for differentiating MS from other white matter lesions, with a pooled sensitivity of 98% and a pooled specificity of 97%. Using individual patient data, the optimal cut-off value for the proportion of lesions with central vein sign on T2*-weighted images was found to be 45%. Although various T2*-weighted images have been used across studies, the current evidence supports the use of the central vein sign on T2*-weighted images to differentiate MS from other white matter lesions.

The differentiation of MS from other white matter lesions can sometimes be challenging, both clinically and radiologically. The proportion of lesions exhibiting the central vein sign is thought to be useful for differentiating MS from some of its mimics^1^. Our results also showed excellent diagnostic performance for differentiating MS from other white matter lesions according to the proportion of lesions exhibiting the central vein sign. In terms of pathophysiology, the inflammatory demyelination in MS spreads in the parenchyma with perivenular extension^41^. However, cerebral small vessel disease is thought to contribute to the chronic ischemic damage presenting at the arteriole^42^, and inflammatory vasculopathies affect medium and small arteries, and are characterized by inflammatory infiltrates of the vessel wall, fibrinoid necrosis, and thrombosis with ischemic change^43^. As the central vein sign is based on a pathological background, the central vein sign may become a promising biomarker for differentiating MS from other white matter lesions.

The current study highlights the fact that the determination of an optimal cut-off value for the proportion of lesions with a central vein sign on T2*-weighted images is clinically and radiologically important if standardized T2*-weighted images are to be used in daily clinical practice. We found that individual cut-off values ranged from 30% to 67%, and that the optimal cut-off value using individual patient data was 45%, resulting in a sensitivity of 97% and specificity of 99%. Although our results were outstanding, the application of this optimal cut-off value requires time-consuming lesion counting and frequency estimation, which may be difficult to conduct in daily clinical practice. A recent study showed the possibility of a fully automated method for detecting the central vein sign, demonstrating a promising performance^44^. However, further studies are needed to validate fully automated methods for detecting the central vein sign.

The North American Imaging in Multiple Sclerosis Cooperative mentioned that imaging of veins in the brain can be performed using T2*-based MRI sequences at any magnetic field strength (1.5, 3, or 7-Tesla)^5^. In addition, high-resolution isotropic T2*-weighted 3D EPI is currently the most promising sequence, and FLAIR* has the potential to become a standard clinical protocol^5^. However, these sequences have not been widely used because of the difficulty in optimizing protocols and post-processing. Therefore, standardization of T2*-weighted imaging is crucial. We found that five studies using FLAIR* demonstrated excellent diagnostic performance for diagnosing MS^7,10,12,20,24^. To generate FLAIR* images, co-registration, interpolation, and multiplication processes are needed^35^. For widespread dissemination of FLAIR*, manufacturer-provided software for direct automated image post-processing on the scanner is necessary.

Although our study results showed the area under the HSROC curve of 0.99 for diagnosing MS using the central vein sign, there are several issues should be considered. Our study is vulnerable to inclusion bias because of the selection of controls. Various comparison groups such as small vessel disease, NMOSD, CNS inflammatory vasculopathies, healthy controls, and non-MS white matter lesions were included. In addition, a previous study showed that the specificity for diagnosing MS using brain MRI with American Academy of Neurology criteria was only 29%, which indicated an increased risk of false-positive diagnosis of MS^45^. Therefore, careful clinical application should be made using our results in daily clinical practice.

This study has several limitations. First, only 6 of 21 eligible studies were of a prospective design^8,10,15,18,21,23^ and 20 studies were single institution studies. In addition, patient number of the included studies were relatively small (median 20, range, 5–68). Second, 13 of 21 eligible studies were case-control designs, which are vulnerable to selection bias^6–9,12,14–17,21,23,24,26^. Third, publication bias was present across the included studies (*p* < 0.01). Therefore, there may be a possibility that the diagnostic performance of central vein sign on T2*-weighted images for diagnosing MS may be overestimated. Last but not least, although all studies used T2*-based MRI sequences, various T2*-weighted images including SWI or FLAIR*, or T2*-weighted images were used. However, all the eligible studies represented the full extent of the currently available evidence. To overcome these limitations, we conducted this systematic review and meta-analysis using recent robust methodology, including hierarchical logistic regression modeling^30–32^, and reported this study according to prestigious guidelines such as the Handbook for Diagnostic Test Accuracy Reviews published by the Cochrane Collaboration^46^, the Agency for Healthcare Research and Quality (AHRQ)^47^, and PRISMA^48^.

In conclusion, although various T2*-weighted images have been used across studies, the current evidence supports the use of the central vein sign on T2*-weighted images to differentiate MS from other white matter lesions.

## Supplementary information

Supplementary materials
