# Supplementary material for: The “Central Vein Sign” on T2*-weighted Images as a Diagnostic Tool in Multiple Sclerosis: A Systematic Review and Meta-analysis using Individual Patient Data
Source: Sci Rep. 2019 Dec 3;9:18188. doi: 10.1038/s41598-019-54583-3 (PMC6890741; doi:10.1038/s41598-019-54583-3)
Supplement: Supplementary file 1 — Supplementary materials [file 41598_2019_54583_MOESM1_ESM.docx]

**The “Central Vein Sign” on T2*-weighted Images as a Diagnostic Tool in Multiple Sclerosis: A Systematic Review and Meta-analysis using Individual Patient Data**

Chong Hyun Suh, M.D.^1^, Sang Joon Kim, M.D., Ph.D.^1^, Seung Chai Jung, M.D., Ph.D.^1^, Choong Gon Choi, M.D., Ph.D.^1^, Ho Sung Kim, M.D., Ph.D.^1^

^1^Department of Radiology and Research Institute of Radiology, University of Ulsan College of Medicine, Asan Medical Center, 86 Asanbyeongwon-Gil, Songpa-Gu, Seoul 05505, Republic of Korea

**Supplementary Materials**

***Literature Search***

The details of the study selection process are illustrated in Figure 1. The literature search found 197 articles. After removing 12 duplicate articles, screening of the 185 remaining titles and abstracts was conducted. From these, a further 155 articles were excluded. Full-text reviews of the 30 remaining potentially eligible articles were performed, and a further nine studies were excluded for the following reasons: partially overlapping patient cohorts (n = 4) [1-4], studies not in the field of interest (n = 3) [5-7], a study for which a study outcome could not be attained (n = 1) [8], and an animal study (n = 1) [9]. Finally, 21 eligible articles encompassing 501 patients with MS were included in the analyses.

***Quality Assessment***

The quality of the 21 eligible studies was considered as moderate, with more than four of the seven domains being satisfied (Supplementary Figure 1). In terms of patient selection, 18 studies were considered to have an unclear risk of bias because patient enrollment was not reported [10-27]. In addition, 13 studies were considered to have a high risk of bias because of a case-control design [10-13,16,18-21,23,25-27]. In terms of the index domain, seven studies were considered to have an unclear risk of bias because it was unclear whether interpretation of the central vein sign was performed in a blinded fashion [10,14,15,17,18,22,24]. In terms of the reference standard domain, four studies were considered to have an unclear risk of bias because they did not mention the detailed criteria for MS diagnosis [15,28,22,24].

**Supplementary Figure 1.** Quality Assessment of Diagnostic Accuracy Studies-2 (QUADAS-2) criteria for the 21 eligible studies. The grouped bar charts demonstrate the risk of bias and concerns on the applicability.

**
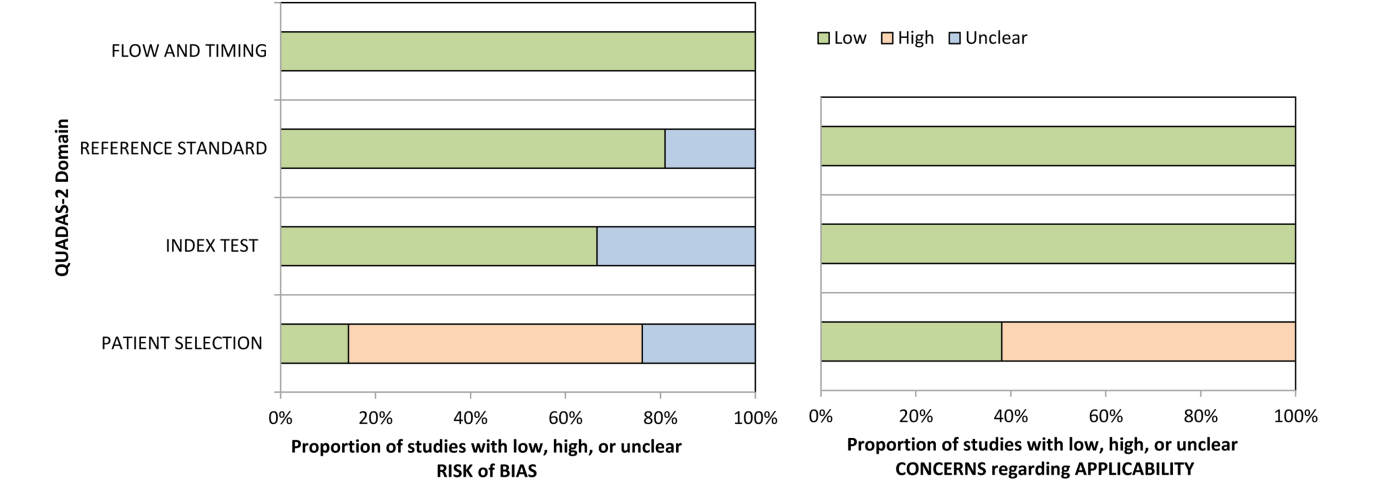
**

**Supplementary Figure 2.** Funnel plots of the pooled incidence of the central vein sign on T2*-weighted images of patients with MS. The funnel plots revealed an absence of publication bias (*p* = 0.63).

**
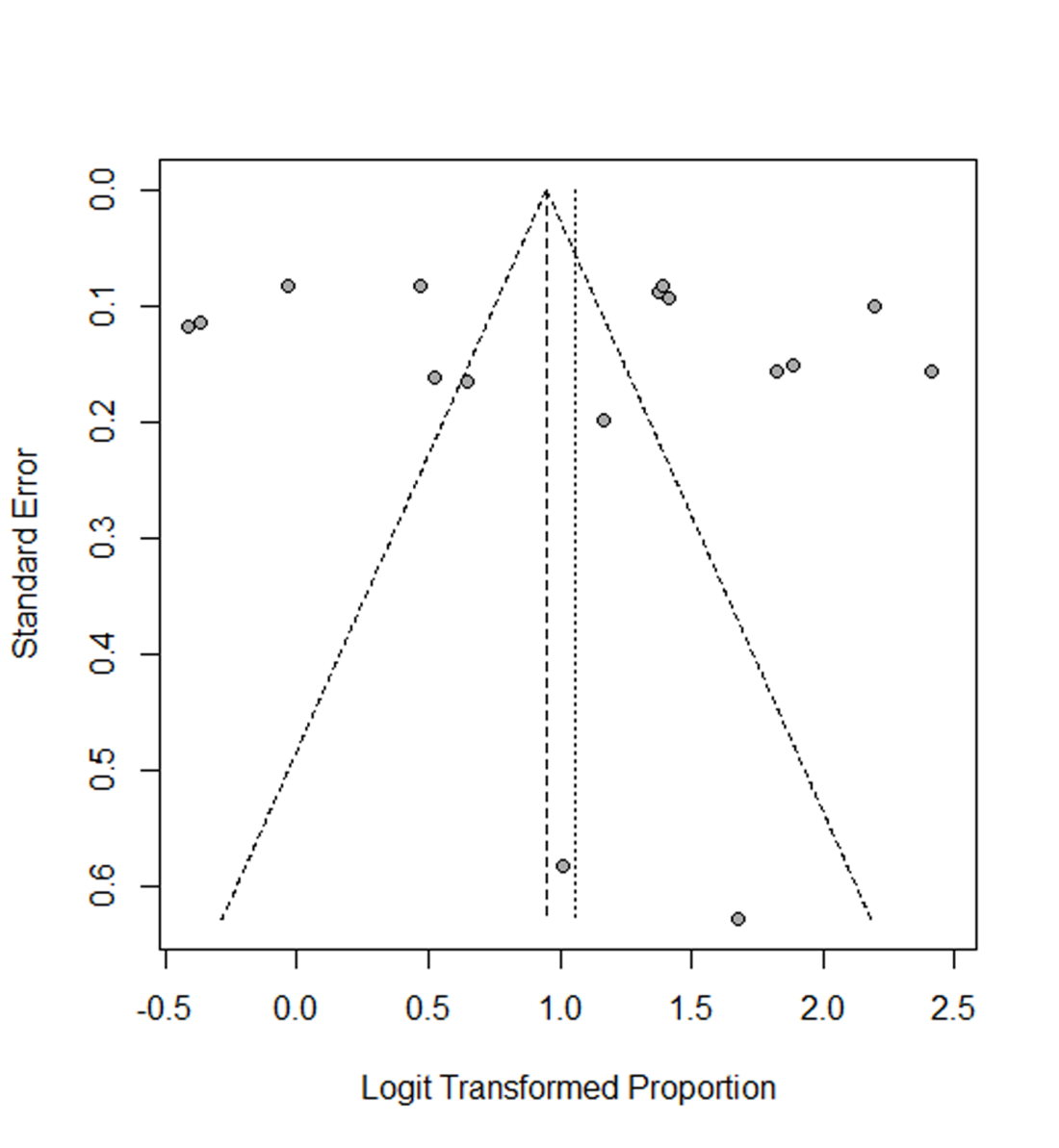
**

**Supplementary Figure 3.** Deeks’ funnel plot used to evaluate potential publication bias. Publication bias was present.


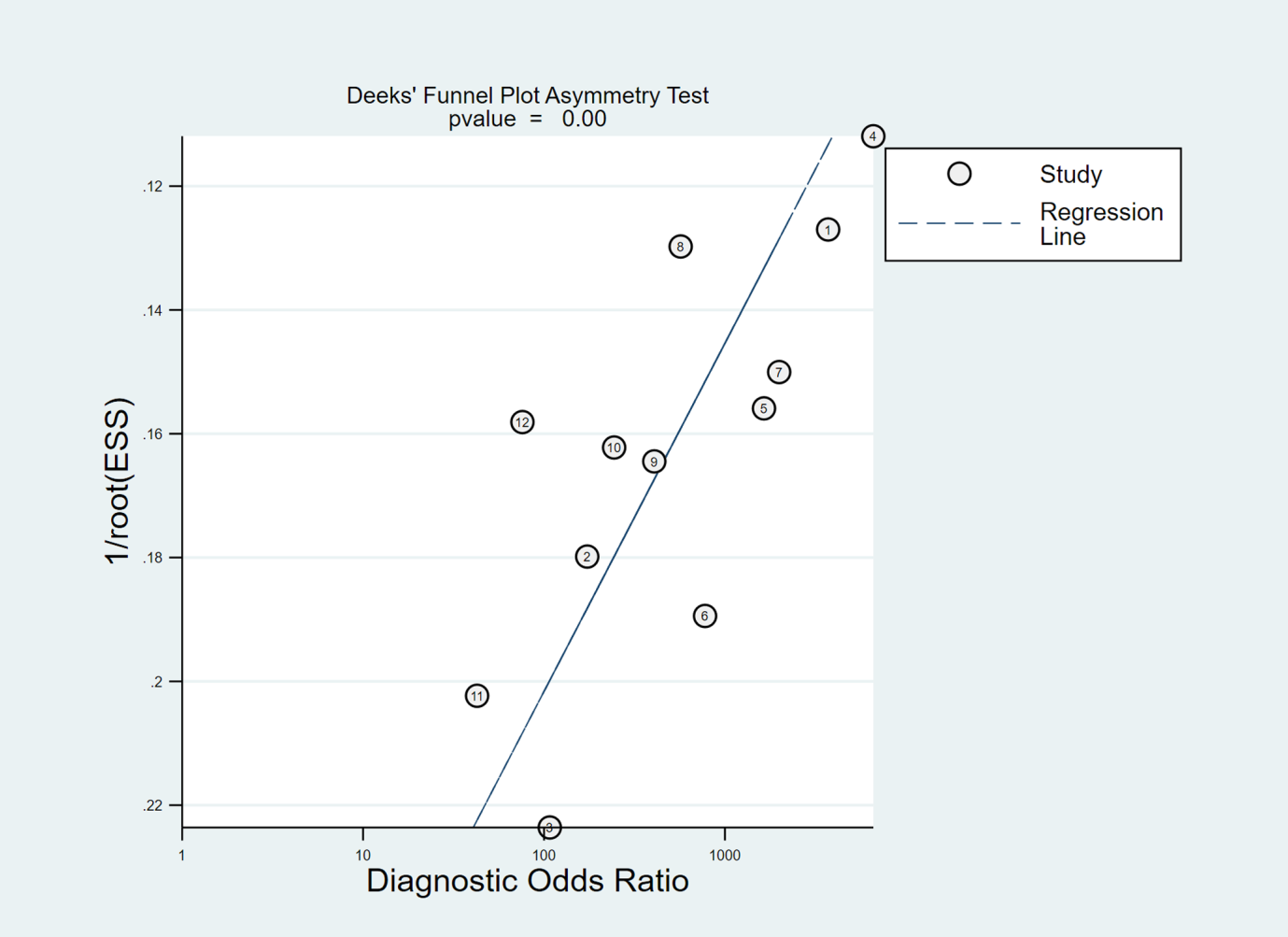


**Reference**

1. Behrens JR, Wanner J, Kuchling J et al (2018) 7 Tesla MRI of Balo's concentric sclerosis versus multiple sclerosis lesions. Annals of Clinical and Translational Neurology 5:900-912. doi:10.1002/acn3.572

2. Dworkin JD, Sati P, Solomon A et al (2018) Automated Integration of Multimodal MRI for the Probabilistic Detection of the Central Vein Sign in White Matter Lesions. AJNR American journal of neuroradiology 39:1806-1813. doi:10.3174/ajnr.A5765

3. Samaraweera AP, Clarke MA, Whitehead A et al (2017) The Central Vein Sign in Multiple Sclerosis Lesions Is Present Irrespective of the T2* Sequence at 3 T. Journal of neuroimaging : official journal of the American Society of Neuroimaging 27:114-121. doi:10.1111/jon.12367

4. Wuerfel J, Sinnecker T, Ringelstein EB et al (2012) Lesion morphology at 7 Tesla MRI differentiates Susac syndrome from multiple sclerosis. Multiple sclerosis (Houndmills, Basingstoke, England) 18:1592-1599. doi:10.1177/1352458512441270

5. Absinta M, Nair G, Sati P, Cortese IC, Filippi M, Reich DS (2015) Direct MRI detection of impending plaque development in multiple sclerosis. Neurology(R) neuroimmunology & neuroinflammation 2:e145. doi:10.1212/nxi.0000000000000145

6. Rogers DM, Shah LM, Wiggins RH, 3rd (2018) The Central Vein: FLAIR Signal Abnormalities Associated with Developmental Venous Anomalies in Patients with Multiple Sclerosis. AJNR American journal of neuroradiology 39:2007-2013. doi:10.3174/ajnr.A5819

7. Sinnecker T, Bozin I, Dorr J et al (2013) Periventricular venous density in multiple sclerosis is inversely associated with T2 lesion count: a 7 Tesla MRI study. Multiple sclerosis (Houndmills, Basingstoke, England) 19:316-325. doi:10.1177/1352458512451941

8. Sati P, George IC, Shea CD, Gaitan MI, Reich DS (2012) FLAIR*: a combined MR contrast technique for visualizing white matter lesions and parenchymal veins. Radiology 265:926-932. doi:10.1148/radiol.12120208

9. Gaitan MI, Maggi P, Wohler J et al (2014) Perivenular brain lesions in a primate multiple sclerosis model at 7-tesla magnetic resonance imaging. Multiple sclerosis (Houndmills, Basingstoke, England) 20:64-71. doi:10.1177/1352458513492244

10. Al-Zandi SH, Fayadh NAH, Al-Waely NKN (2018) Central vein sign detected by SWI at 3T MRI as a discriminator between multiple sclerosis and leukoaraiosis. Egyptian Journal of Radiology and Nuclear Medicine 49:158-164. doi:10.1016/j.ejrnm.2017.09.003

11. Campion T, Smith RJP, Altmann DR et al (2017) FLAIR* to visualize veins in white matter lesions: A new tool for the diagnosis of multiple sclerosis? European radiology 27:4257-4263. doi:10.1007/s00330-017-4822-z

12. Cortese R, Magnollay L, Tur C et al (2018) Value of the central vein sign at 3T to differentiate MS from seropositive NMOSD. Neurology 90:e1183-e1190. doi:10.1212/wnl.0000000000005256

13. Darwish EAF, Ibrahim YA, Zamzam DA (2018) Value of central vein sign in discriminating multiple sclerosis plaques from other white matter lesions. Egyptian Journal of Radiology and Nuclear Medicine 49:165-171. doi:10.1016/j.ejrnm.2017.12.008

14. Gabr RE, Pednekar AS, Kamali A et al (2018) Interleaved susceptibility-weighted and FLAIR MRI for imaging lesion-penetrating veins in multiple sclerosis. Magnetic resonance in medicine 80:1132-1137. doi:10.1002/mrm.27091

15. Gaitan MI, de Alwis MP, Sati P, Nair G, Reich DS (2013) Multiple sclerosis shrinks intralesional, and enlarges extralesional, brain parenchymal veins. Neurology 80:145-151. doi:10.1212/WNL.0b013e31827b916f

16. George IC, Sati P, Absinta M et al (2016) Clinical 3-tesla FLAIR* MRI improves diagnostic accuracy in multiple sclerosis. Multiple sclerosis (Houndmills, Basingstoke, England) 22:1578-1586. doi:10.1177/1352458515624975

17. Grabner G, Dal-Bianco A, Schernthaner M, Vass K, Lassmann H, Trattnig S (2011) Analysis of multiple sclerosis lesions using a fusion of 3.0 T FLAIR and 7.0 T SWI phase: FLAIR SWI. Journal of magnetic resonance imaging : JMRI 33:543-549. doi:10.1002/jmri.22452

18. Hosseini Z, Matusinec J, Rudko DA et al (2018) Morphology-Specific Discrimination between MS White Matter Lesions and Benign White Matter Hyperintensities Using Ultra-High-Field MRI. AJNR American journal of neuroradiology 39:1473-1479. doi:10.3174/ajnr.A5705

19. Kau T, Taschwer M, Deutschmann H, Schonfelder M, Weber JR, Hausegger KA (2013) The "central vein sign": is there a place for susceptibility weighted imaging in possible multiple sclerosis? European radiology 23:1956-1962. doi:10.1007/s00330-013-2791-4

20. Lamot U, Avsenik J, Sega S, Surlan Popovic K (2017) Presence of central veins and susceptibility weighted imaging for evaluating lesions in multiple sclerosis and leukoaraiosis. Multiple sclerosis and related disorders 13:67-72. doi:10.1016/j.msard.2017.02.008

21. Lane JI, Bolster B, Campeau NG, Welker KM, Gilbertson JR (2015) Characterization of multiple sclerosis plaques using susceptibility-weighted imaging at 1.5 T: can perivenular localization improve specificity of imaging criteria? Journal of computer assisted tomography 39:317-320. doi:10.1097/rct.0000000000000233

22. Luo J, Yablonskiy DA, Hildebolt CF, Lancia S, Cross AH (2014) Gradient echo magnetic resonance imaging correlates with clinical measures and allows visualization of veins within multiple sclerosis lesions. Multiple sclerosis (Houndmills, Basingstoke, England) 20:349-355. doi:10.1177/1352458513495935

23. Mistry N, Abdel-Fahim R, Samaraweera A et al (2016) Imaging central veins in brain lesions with 3-T T2*-weighted magnetic resonance imaging differentiates multiple sclerosis from microangiopathic brain lesions. Multiple sclerosis (Houndmills, Basingstoke, England) 22:1289-1296. doi:10.1177/1352458515616700

24. Oztoprak B, Oztoprak I, Yildiz OK (2016) The effect of venous anatomy on the morphology of multiple sclerosis lesions: a susceptibility-weighted imaging study. Clinical radiology 71:418-426. doi:10.1016/j.crad.2016.02.005

25. Sinnecker T, Dorr J, Pfueller CF et al (2012) Distinct lesion morphology at 7-T MRI differentiates neuromyelitis optica from multiple sclerosis. Neurology 79:708-714. doi:10.1212/WNL.0b013e3182648bc8

26. Solomon AJ, Watts R, Ontaneda D, Absinta M, Sati P, Reich DS (2018) Diagnostic performance of central vein sign for multiple sclerosis with a simplified three-lesion algorithm. Multiple sclerosis (Houndmills, Basingstoke, England) 24:750-757. doi:10.1177/1352458517726383

27. Tallantyre EC, Morgan PS, Dixon JE et al (2009) A comparison of 3T and 7T in the detection of small parenchymal veins within MS lesions. Investigative radiology 44:491-494. doi:10.1097/RLI.0b013e3181b4c144

28. Lummel N, Boeckh-Behrens T, Schoepf V, Burke M, Bruckmann H, Linn J (2011) Presence of a central vein within white matter lesions on susceptibility weighted imaging: a specific finding for multiple sclerosis? Neuroradiology 53:311-317. doi:10.1007/s00234-010-0736-z
